# POLICY SERIES: ELECTION YEAR RESULTS: IMPLICATIONS FOR OUR FUTURE

**DOI:** 10.1093/geroni/igae098.1389

**Published:** 2024-12-31

**Authors:** Brian Lindberg, Patricia D’Antonio

**Affiliations:** Chair; Discussant

## Abstract

Will Social Security be targeted? Will Obamacare be gutted? How will Medicare drug prices be affected? These and many other programs and policies will be influenced by who sits in the White House and the Congress starting in 2025. Join your colleagues and GSA’s own pundits for a discussion on what went down on November 5th and what will be coming up in aging policy.

